# Suppression of FVIII-Specific Memory B Cells by Chimeric BAR Receptor-Engineered Natural Regulatory T Cells

**DOI:** 10.3389/fimmu.2020.00693

**Published:** 2020-04-21

**Authors:** Alessandra De Paula Pohl, Shivaprasad H. Venkatesha, Ai-Hong Zhang, David W. Scott

**Affiliations:** Department of Medicine, Uniformed Services University of the Health Sciences, Bethesda, MD, United States

**Keywords:** FVIII, memory B cells, regulatory T cells, chimeric receptor, B-cell antibody receptor

## Abstract

Anti-drug antibody formation poses tremendous obstacles for optimal treatment of hemophilia A (HA). In this study, we sought to utilize chimeric receptor-modified natural regulatory T cells (Tregs) to target FVIII-specific memory B cells, which are responsible for persistent anti-FVIII neutralizing antibodies (inhibitors) in HA patients. Thus, CD4^+^CD25^*hi*^CD304^+^ natural Tregs were FACS sorted from naïve C57BL/6 mice and retrovirally transduced to express a chimeric B-cell antibody receptor (BAR) containing the immunodominant A2 domain of FVIII. Plasmablast-depleted (CD138^*neg*^) splenocytes from FVIII immunized FVIII-knockout HA mice served as the source for FVIII-specific memory B cells, which were specifically stimulated *in vitro* with FVIII and enumerated in a B-cell ELISPOT assays. Adding A2-BAR Tregs (1 per 150 splenocytes), but not conventional T cells, to the CD138^–^ splenocytes significantly suppressed the formation of anti-FVIII antibody secreting cells (ASC), compared to the non-relevant OVA-BAR Tregs control group. The observation that A2-BAR Tregs can suppress the response to FVIII suggests that bystander suppression can occur in the local milieu in this system. Transwell experiments confirmed that the suppression was contact-dependent. Moreover, even in the presence of antibodies to FVIII (so-called inhibitors), similarly prepared CD4^+^CD25^*hi*^CD127^*low*^ A2-BAR *human* natural Tregs completely suppressed polyclonal anti-FVIII ASC formation. In conclusion, we demonstrated *in vitro* that FVIII domain-expressing BAR Tregs could efficiently target and suppress FVIII-specific memory B cells.

## Introduction

Hemophilia A (HA) is a hereditary bleeding disorder, caused by mutations in the *F8* gene encoding pro-coagulant factor VIII (FVIII) (1). Despite great improvement in the management of the disease, one remaining major issue is the formation of anti-FVIII neutralizing antibodies (inhibitors), which occur in up to 30% of severe HA and about 5% of moderate and mild HA patients (2). Currently, the only clinically proven strategy to eradicate the inhibitors is called immune tolerance induction therapy (ITI). First described 40 years ago (3), ITI features repeated, high dose FVIII infusions until the inhibitor becomes undetectable. The mechanism of action for ITI remains incompletely understood. Clinical evidence suggests that FVIII-specific memory B cells were deleted in HA patients that had successfully completed ITI (4). Indeed, FVIII-specific memory B cells were suppressed in the presence of high dose FVIII *in vitro* and *in vivo* using murine HA models (5–7). Although ITI can eradicate inhibitors in about 60–80% of eligible patients, some patients undergo ITI for up to 3 years, and this therapy is extremely expensive. ITI failures necessitate alternative approaches, which may not be as effective in restoring hemostasis as FVIII in some settings, e.g., trauma or surgery. Therefore, restoring tolerance to FVIII is an unmet need (2).

We have previously reported the approach of targeting pathogenic B cells using antigen-specific regulatory T cells (Tregs) or CD8 T cells (8, 9). Analogous to chimeric antigen receptor (CAR) technology that has been successfully used in cancer immunotherapy (10), we developed a chimeric receptor comprising a protein domain antigen linked to transmembrane and intracellular signaling domains CD28-CD3ζ. We termed this a B-cell antibody receptor, or “BAR”. Adoptive transfer of a combination of FVIII A2 domain-BAR transduced human Tregs and FVIII C2 domain-BAR transduced human Tregs completely prevented the anti-FVIII antibody formation in response to FVIII/IFA immunization of HA mice (8). Because FVIII contains multiple domains, it is not known if engineered Tregs expressing BARs consisting of single domains will be sufficient to suppress the production of polyclonal anti-FVIII antibodies specific for different epitopes of FVIII. Furthermore, it is known that Tregs can impose suppression over a variety of cell types. Several studies have already indicated direct suppression/killing of B cells by CD4^+^CD25^+^ Tregs (11–15), which begs the question whether antigen-specific Tregs, such as chimeric BAR receptor engineered natural Tregs, could be utilized to suppress the activity of FVIII-specific memory B cells.

In this study, we addressed the above questions by using plasmablast-depleted (CD138^–^) splenocytes from FVIII immunized HA mice as the source for FVIII-specific memory B cells. The suppressive effect of mouse A2 domain-BAR natural Tregs on the activity of polyclonal FVIII-specific memory B cells was determined *in vitro* using a B-cell ELISPOT assay. In addition, the *in vitro* suppression assay was confirmed by using A2 domain-BAR transduced human Tregs in the same assay, in the presence/absence of neutralizing anti-FVIII antibodies (inhibitors).

## Materials and Methods

### Mice and FVIII Immunization

E16 mice (*F8* exon 16 knockout) on a C57BL/6 background were originally from the colony of Dr. L. Hoyer at the American Red Cross (16, 17). Male and homozygous female E16 mice were maintained in the vivarium of Uniformed Services University of the Health Sciences (USUHS), and were immunized by weekly intravenous injections of 1 μg recombinant human FVIII (rFVIII) in 100 μl PBS for at least 4 weeks to allow the generation of FVIII-specific memory B cells. In some experiments, the immunization was done subcutaneously with a single injection of 2 μg rFVIII emulsified in Incomplete Freund’s Adjuvant. The presence of high-titer anti-FVIII antibodies and high-titer inhibitors was confirmed by a FVIII ELISA and a modified Bethesda assay, respectively, as previously described (18). Naïve C57BL/6 mice were purchased from the Jackson laboratory and served as the donors of Tregs for engineering to make BAR-Tregs. Animal procedures were approved by the Institutional Animal Care and Use Committee at USUHS.

### Reagents

Recombinant human IL-2 (rIL-2) was provided by the National Cancer Institute Biological Resources Branch (Frederick, MD, United States). Recombinant human FVIII (rFVIII) was provided by Baxalta, Inc. (Vienna, Austria). An anti-FVIII A2 mAb (4A4) was a gift from Dr. Pete Lollar at Emory University. The following commercial anti-mouse antibodies were used either for stimulating T cells or for flow cytometry: anti-CD3ε (145-2C11), anti-CD28 (37.51), FITC anti-CD4 (GK1.5), PE anti-CD25 (PC61), PE-Cy7 anti-CD304 (3E12), PE anti-Helios (22F6), Pacific Blue anti-Granzyme B (GB11), PerCP-Cy5.5 anti-IL 10 (JES5-16E3), PerCP-Cy5.5 anti-TGF-β1 (TW7-16B4) from BioLegend; APC anti-Foxp3 (FJK-16s) from eBioscience. Rabbit anti-OVA IgG was purchased from Organon Teknika Corp (West Chester, PA, United States). CD4 (L3T4) microbeads (Miltenyl Biotec) was used to positively select mouse CD4^+^ T cells.

### Construction of BAR Retroviral Vectors

**FIGURE 1:**
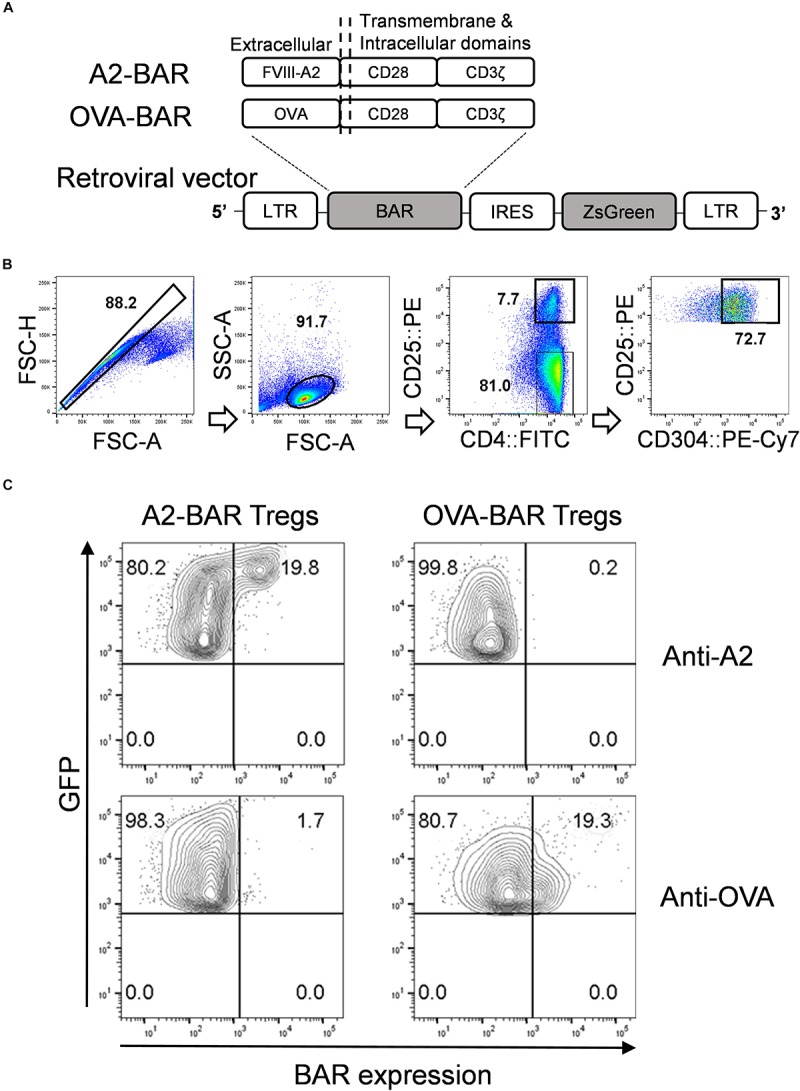
Generation of mouse CD4^+^CD25^*hi*^CD304^+^ natural Tregs expressing the chimeric BAR receptor. **(A)** Schematic illustration for the retroviral constructs for FVIII A2-BAR and control OVA-BAR. **(B)** Gating strategy for FACS sorting of mouse natural Tregs. **(C)** Surface BAR expression on transduced mouse Tregs. FACS sorted and activated mouse natural Tregs were transduced with retroviral supernatant for either FVIII A2-BAR or the control OVA-BAR. Five days following the transduction, the cells were surface stained with either anti-A2 (mAb 4A4) or anti-OVA (rabbit anti-OVA IgG), followed by a fluorescence labeled 2nd antibody. The cells were gated on singlets → size → live and GFP^+^.

Construction of BAR retroviral vectors containing the FVIII A2 or chicken Ovalbumin (OVA) was as described (8). Briefly, the cDNA sequence encoding the human FVIII A2 domain or chicken OVA were obtained from GenBank. As illustrated in Figure 1A, each of these two cDNA sequences was linked via a G4S sequence to downstream CD28-CD3ζ transmembrane and intracellular signaling domains. The constructed DNA sequence for BARs was codon optimized and synthesized by GenScript (Piscataway, NJ, United States), and inserted into a pRetroX-IRES-ZsGreen1 (Clontech Laboratories, Mountain View, VA, United States) retroviral vector. The retroviral particles were produced using a Phoenix-Eco packaging system (Clontech Laboratories). Culture supernatants containing the retroviral particles were aliquoted and stored at −80°C until use.

### Isolation of Mouse T Cells and Transduction

On day 0, spleens were isolated from naïve 6–8 week old C57BL/6 mice, and single cell suspensions of splenocytes were prepared after red blood cell lysis. The cells were first enriched for CD4^+^ T cells using magnetic cell sorting (MACS) and then further purified to isolate natural Tregs (CD4^+^CD25^*hi*^CD304^+^) and conventional T cells (Tcon, CD4^+^CD25^–^) by sorting on a FACSAria II cell sorter (BD Biosciences, Franklin Lakes, NJ) (Figure 1B). For Treg sorting, the purity of CD4^+^CD25^*hi*^CD304^+^ gate was 96.9 ± 0.4%. The sorted cells were cultured in complete RPMI 1640 culture medium supplemented with 10% FBS, 1 mM sodium pyruvate, 10 mM HEPES buffer, 2 mM L-glutamine, 50 U/ml Penicillin-Streptomycin, and 50 μM 2-Mercaptoethanol in the presence of 200 U/ml rIL-2.

FACS-sorted Tregs or Tcon were stimulated with plate-bound anti-mouse CD3ε, in the presence of 2 μg/ml soluble anti-mouse CD28 and 200 U/ml rIL-2 for 48 h. Transduction was performed on day 3 by adding the retroviral particle supernatant to a 10 μg/ml Retronectin (Clontech) pretreated culture plate and spinning it at 2000 × *g*, 32°C for 2 h, followed by centrifugation of the activated T cells onto the viral particle-coated plate at 500 × *g*, 32°C for 15 min. The cells were split every 2 or 3 days with complete culture medium containing 200 U/ml rIL-2. Five days after transduction, the BAR-transduced Tregs and Tcon cells were FACS sorted based on GFP expression. The sorted cells were cultured in complete RPMI culture medium in the absence of added rIL-2 for 24 h, before been used in the suppression assays.

### Generation of Human BAR Natural Tregs

Human FVIII A2 domain-BAR Tregs were prepared as described (8). All procedures using human blood samples were approved by the Uniformed Services University of the Health Sciences Institutional Review Board.

### FACS Staining

The cells (1 × 10^6) were stained with the indicated antibodies together with a fixable viability dye eFluor 780 (eBioscience). The cells were then fixed with 2% Formaldehyde in PBS containing 0.02% Tween 20 at 37°C for 10 min. Data were then acquired on an LSR II instrument (BD) and analyzed using FlowJo software (Tree Star, Ashland, OR, United States).

For intracellular staining, the cells were fixed and then permeabilized overnight in 0.02% Triton-X 100 in PBS containing 1% FBS, followed by staining with the indicated antibodies for 4 h at 4°C. The cells were then analyzed as described above.

### *In vitro* Suppression of FVIII-Specific Memory B Cells and B-Cell ELISPOT Assay

Splenocytes from FVIII-immunized HA mice were depleted for CD138^+^ plasmablasts using CD138 microbeads (Miltenyi Biotec), and the resultant pooled CD138^–^ splenocytes served as the source for FVIII-specific memory B cells (5–7). In 48-well culture plates, 6 × 10^6 of CD138^–^ splenocytes were cultured with 40,000 of A2-BAR Tregs or A2-BAR Tcon cells in the presence of 10 ng/ml rFVIII at 37°C for 6 days to promote FVIII-specific memory B cells differentiation into anti-FVIII antibody-secreting cells (ASC). For the B-cell ELISPOT assay, after 6 days the cells were washed twice in culture medium and transferred to 5 μg/ml rFVIII-coated 96-well ELISPOT plates (EMD Millipore) and cultured overnight. The spots indicating FVIII-specific ASCs were visualized through incubation with HRP-rabbit anti-mouse IgG (H + L) (Thermo Fisher Scientific), followed by AEC substrate (BD Biosciences).

### Statistical Analysis

Statistical analysis was performed using Prism software (v6.0; GraphPad Software, La Jolla, CA, United States). A Student’s *t*-test (2-tailed) was chosen to evaluate differences between different groups. A *p*-value < 0.05 was considered statistically significant. Each *in vitro* memory B-cell suppression assay was repeated at least two times, and representative data are shown.

## Results

### FVIII A2-BAR and OVA-BAR Are Expressed on Transduced Mouse Natural Tregs

FVIII A2-BAR was constructed by linking the immunodominant A2 domain of FVIII to the downstream transmembrane and signaling domains, CD28-CD3ζ, via a G_4_S linker. OVA-BAR was constructed similarly and served as the specificity control (Figure 1A).

Transduction efficiencies for A2-BAR and OVA-BAR Tregs were estimated to be 30–70% based on the GFP reporter gene expression (data not shown). After transduction, BAR expression could be detected on the surface of transduced Tregs by staining with specific antibodies against the FVIII-A2 or OVA domains, respectively (Figure 1C).

### BAR Expression Did Not Affect the Suppressive Phenotype of Mouse Natural Tregs

**FIGURE 2:**
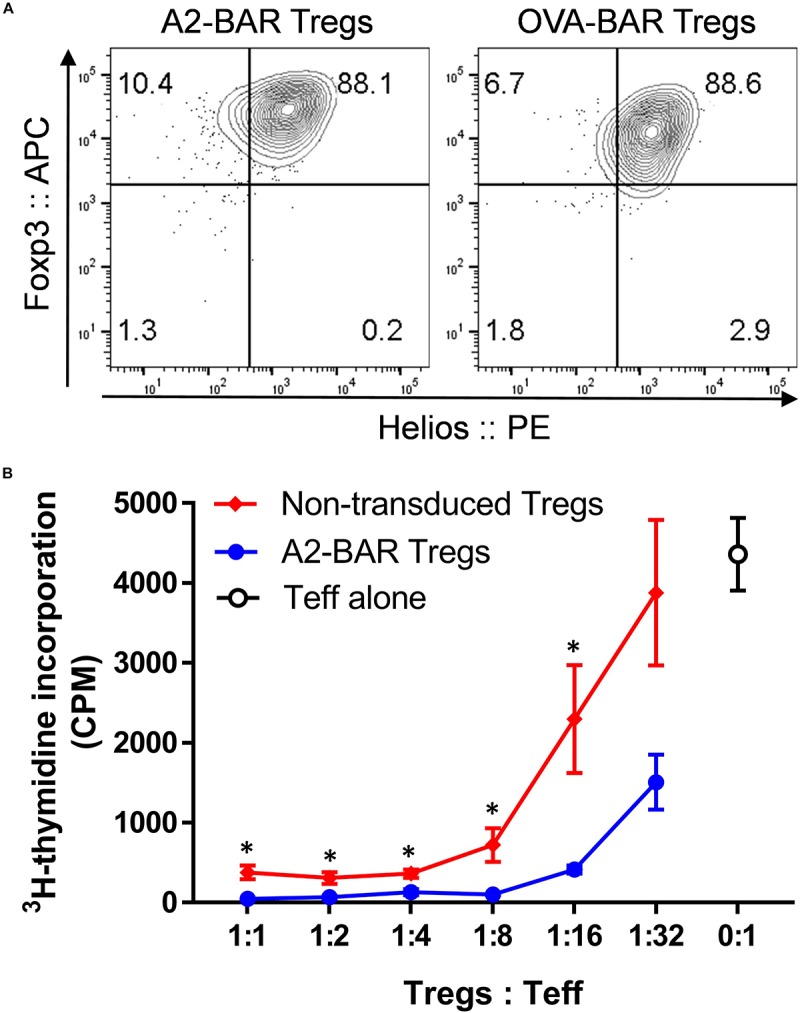
Expression of BAR did not adversely affect the suppressive function of mouse natural Tregs. **(A)** Foxp3 and Helios expression in the prepared mouse BAR Tregs. Five days after the retrovirus mediated transduction, the cells were surface stained with anti-CD4 and fixable viability dye eFluor 780, followed by intracellular staining for Foxp3 and Helios. The cells were gated on singlets → size → live and CD4^+^ → GFP^+^. **(B)** *In vitro* suppression of Tcon proliferation by mouse Tregs. Five days after the A2-BAR retroviral transduction, the mouse Tregs were FACS sorted into A2-BAR Tregs (GFP^+^) and non-transduced Tregs (GFP^–^). For the suppression assay, in 96-well culture plates, 2 × 10^6^ FACS sorted CD4^+^CD25^–^ conventional T cells from naïve E16 mice (Teff) were cultured with either Tregs at various ratios for 3 days, in the presence of 4 × 10^6^ irradiated splenocytes and 2 μg/ml soluble anti-mouse CD3. The cells were then pulsed with 0.5 μCi ^3^H-thymidine for 16 h before readout. The data are expressed as mean ± SEM. *p < 0.05 comparing the A2-BAR Tregs group and the non-transduced Tregs group using a Student’s *t*-test.

To confirm the purity of the BAR Tregs, 5 days following retroviral transduction, the Tregs were stained intracellularly with Treg markers Foxp3 and Helios. As shown in Figure 2A, >95% of the A2-BAR and OVA-BAR Tregs expressed Foxp3, and most of these cells co-expressed Helios, a phenotype consistent with that of natural Tregs (19).

To exclude the possibility that BAR expression could adversely affect Treg functionality, a typical *in vitro* T cell suppression assay was performed. Five days after A2-BAR transduction, mouse Tregs were further FACS sorted into GFP^+^ (A2-BAR Tregs) and GFP^–^ (non-transduced Tregs) fractions, based on the GFP reporter gene expression. Both GFP^+^ A2-BAR Tregs and GFP^–^ non-transduced Tregs were co-cultured with a fixed number of FACS-sorted CD4^+^CD25^–^ conventional T cells (Tcon) at various ratios, in the presence of 2 μg/ml soluble anti-CD3. Similar to the non-transduced mouse Tregs, A2-BAR Tregs dose-dependently suppressed proliferation of Tcon cells. At multiple Tregs/Teff ratios, A2-BAR Tregs were significantly more suppressive than GFP^–^ non-transduced Tregs, indicating that BAR expression did not adversely impact the suppressive quality of the Tregs (Figure 2B).

### FVIII A2-BAR Natural Tregs Contact-Dependently Suppressed FVIII-Specific Memory B Cells

**FIGURE 3:**
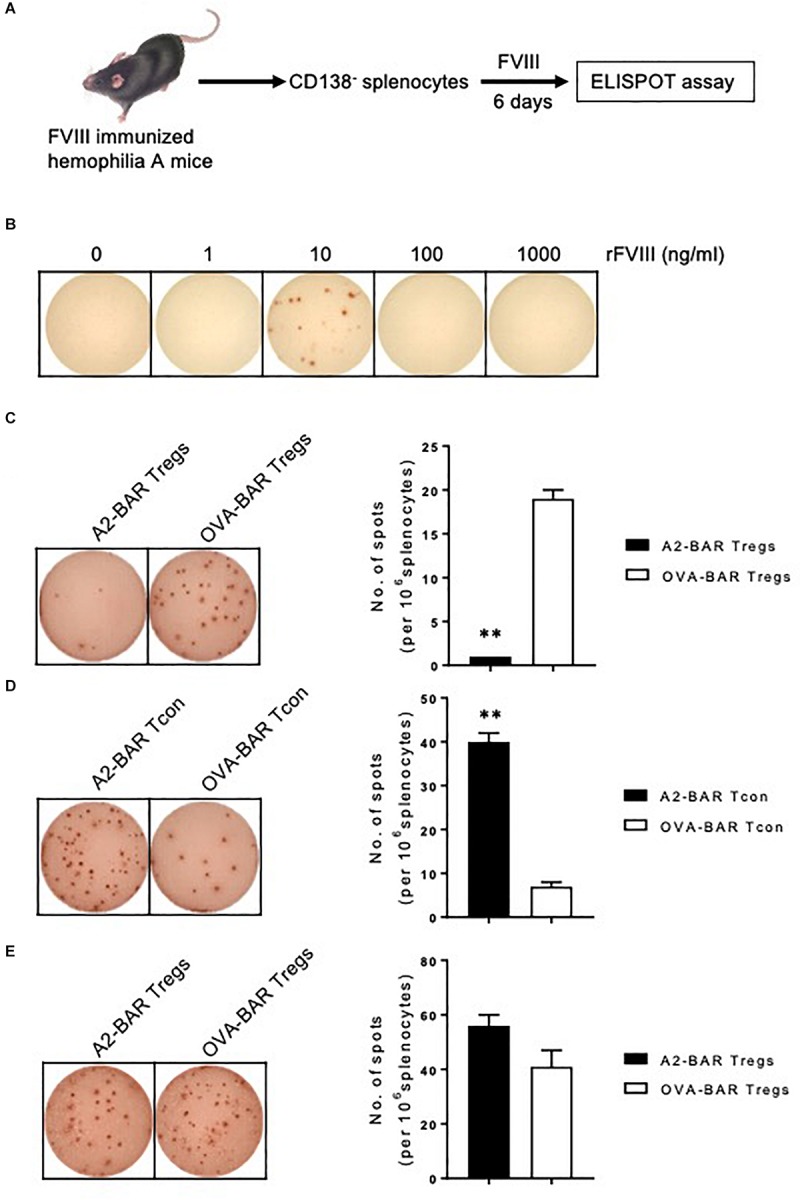
FVIII A2-BAR mouse natural Tregs suppressed FVIII-specific memory B cells *in vitro*. **(A)** Schematic illustration for the detection of FVIII-specific memory B cells. Splenocytes from FVIII-immunized E16 mice were depleted for plasmablasts/plasma cells with CD138 microbeads by MACS. The CD138^–^ splenocytes were cultured in complete culture medium in the presence of optimal amount of rFVIII for 6 days. At the end of the culture, the anti-FVIII antibody secreting cells (ASC), which reflect the number and activity of FVIII-specific memory B cells, were enumerated with a FVIII-specific B-cell ELISPOT assay. **(B)** The effect of rFVIII concentration on the detection of FVIII-specific memory B cells. CD138^–^ splenocytes were cultured in complete culture medium in the presence of increasing amounts of rFVIII for 6 days. The anti-FVIII ASC spots were detected by a FVIII-specific B-cell ELISPOT assay. **(C)** A2-BAR mouse natural Tregs significantly suppressed FVIII-specific memory B cells *in vitro*. In 48-well culture plates, CD138^–^ splenocytes (6 × 10^6^) were co-cultured with 150-fold less (40,000) A2-BAR or OVA-BAR mouse natural Tregs, in the presence of 10 ng/ml rFVIII for 6 days. The anti-FVIII ASC spots were detected by FVIII-specific B-cell ELISPOT assay, and visualized with AEC substrate (BD Bioscience). The ELISPOT plates were analyzed using ImmunoSpot analyzers (CTL Immunospot). **(D)** Expressing A2-BAR on Tcon did not confer suppressive function on FVIII-specific memory B cells. The experiment was performed as described in Figure 3C, except that BAR Tcon cells were used instead of Tregs. **(E)** The suppression of A2-BAR mouse natural Tregs on FVIII-specific memory B cells was contact-dependent. The suppression assay was set up as described in Figure 3C, except a Transwell plate was used. The Tregs were placed in the lower chamber, and the CD138^–^ splenocytes were placed in the upper chamber. No statistical difference was found between the A2-BAR Tregs group and the control OVA-BAR Tregs group. The histograms summarize the data on the left, and the data are expressed as mean ± SEM **(C–E)**. **p < 0.01 between the A2-BAR group and the control OVA-BAR group by the Student’s *t*-test **(C,D)**.

The effect of FVIII A2-BAR Tregs on cognate FVIII-specific memory B cells was tested next. Bicistronic GFP expression was used as a minimal surrogate marker for BAR expression (Figure 1A). The purity of FACS sorted GFP^+^ cells was 96.8 ± 0.5%. FACS sorted GFP^+^ BAR Tregs were used in all the suppression assays described below. Plasma cell-depleted (CD138^–^) splenocytes from FVIII-immunized E16 mice served as the source of FVIII-specific memory B cells (Supplementary Figure S1). As illustrated in Figure 3A, the rare FVIII-specific memory B cells were detected by culturing the splenocytes with FVIII for 6 days, and then by carrying out a FVIII-specific B-cell ELISPOT assay, as previously described (5–7). As shown in Figure 3B, 10 ng/ml rFVIII was the optimal concentration to detect FVIII-specific ASC. A higher concentration of rFVIII suppressed FVIII-specific ASCs formation, which was consistent with previous reports (5–7). Therefore, rFVIII at 10 ng/ml concentration was used for all of the FVIII-specific memory B cell suppression assays.

As shown in Figure 3C, compared to the OVA-BAR Tregs control, adding A2-BAR Tregs significantly suppressed FVIII-specific memory B-cell activity, as reflected by the reduced number of anti-FVIII ASCs (*p* < 0.01) (Figure 3C). The suppressive activity of A2-BAR Tregs on the activity of FVIII-specific memory B cells could not be ascribed to the expression of A2-BAR alone, since the addition of A2-BAR Tcon did not suppress the anti-FVIII ASC formation (Figure 3D).

To address the question of whether cell-cell contact is required for suppression, a transwell culture system was employed to separate the BAR Tregs from the CD138^–^ splenocytes. The suppressive effect of A2-BAR Tregs was completely abolished in the transwell setting, indicating that the suppression of FVIII-specific memory B cells was contact-dependent (Figure 3E).

### Cytokine Expression by the Activated BAR Mouse Natural Tregs

**FIGURE 4:**
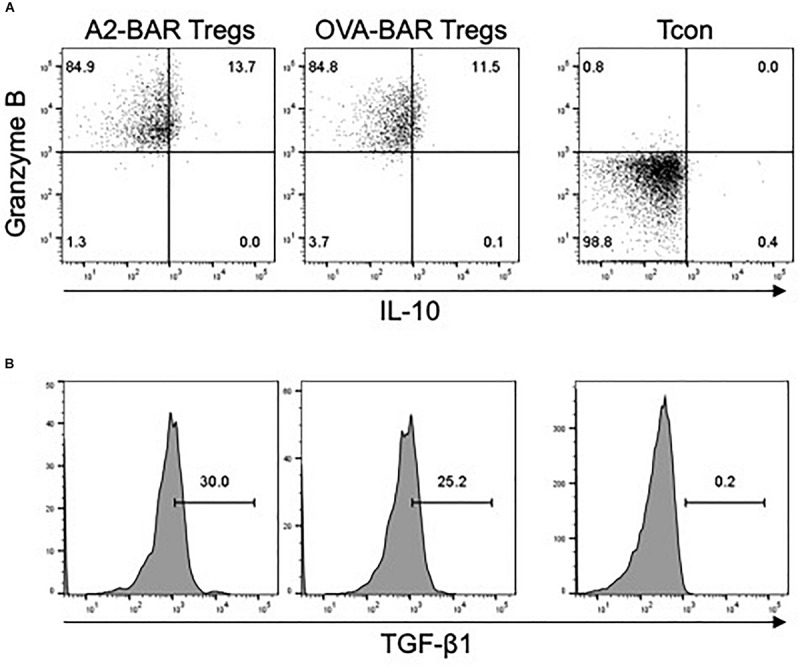
Cytokine expression by mouse BAR natural Tregs. Five days after the retrovirus-mediated transduction, the Tregs were rested in medium in the absence of IL-2 overnight, and then stimulated for 5 h with cell stimulation cocktail containing PMA, Ionomycin, Monensin and Brefeldin A. The cells were then surface stained with anti-CD4 and fixable viability dye eFluor 780, followed by intracellular staining for Granzyme B, IL-10, or TGF-β1. Freshly isolated CD4^+^CD25^–^ mouse conventional T cells (Tcon) was stimulated the same way and served as a control. **(A)** The expression of Granzyme B and IL-10 in the mouse BAR natural Tregs. **(B)** The expression of TGF-β1 in the activated BAR Tregs. The cells shown were gated on singlets → size → viable CD4^+^ → GFP^+^.

Next, we examined the expression of several important cytokines, including IL-10, TGF-β1, and Granzyme B, which could potentially play a role in the suppressive function of the BAR Tregs. Compared with the freshly isolated mouse Tcon, slight upregulation of IL-10 and TGF- β1 expression could be detected in the activated BAR mouse Tregs. Strikingly, >96% of the BAR Tregs expressed Granzyme B (Figure 4). The exact roles of these effector cytokines during the suppression of FVIII-specific memory B cells by BAR mouse natural Tregs are to be further investigated.

### Human FVIII A2-BAR Tregs Suppressed Murine FVIII-Specific Memory B Cells in the Presence of Murine Inhibitory Antibodies

**FIGURE 5:**
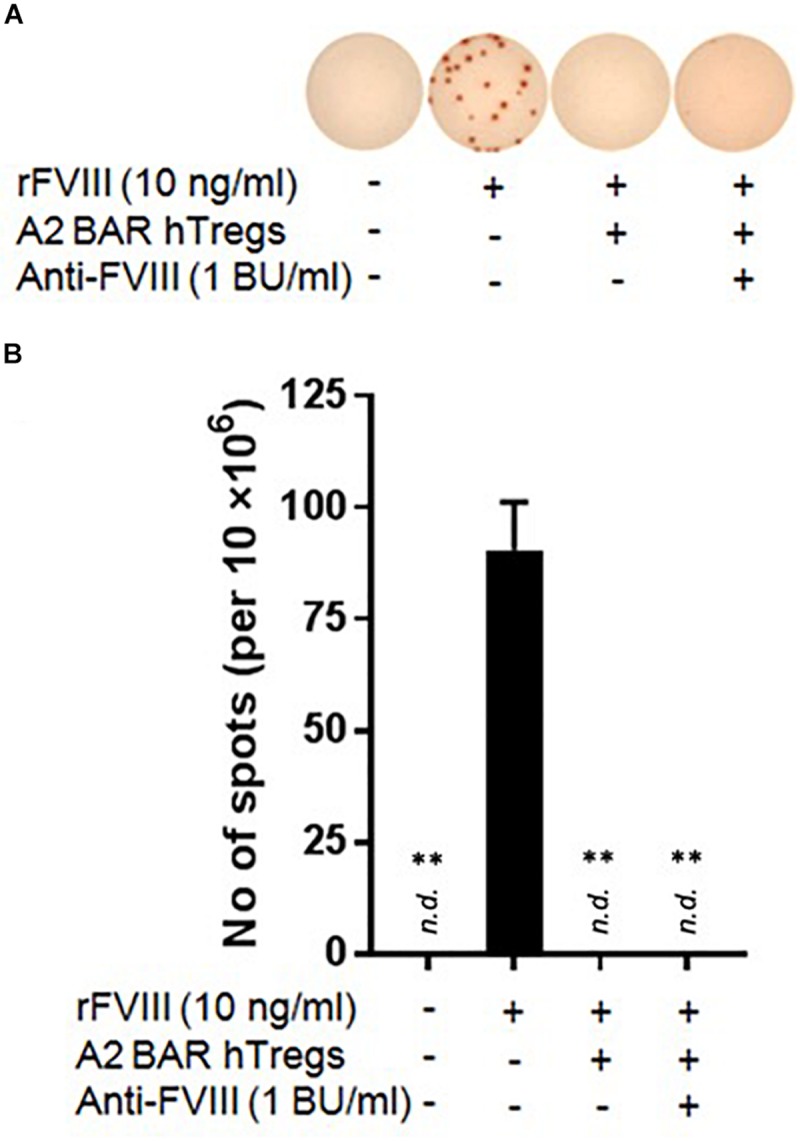
Human FVIII A2-BAR Tregs suppressed murine FVIII-specific memory B cells in the presence of low-titer inhibitors. In 48-well culture plates, CD138^–^ splenocytes (4 × 10^6^) were co-cultured with A2-BAR human Tregs (1 × 10^6^) in the presence/absence of rFVIII for 6 days. In one group, anti-FVIII mouse sera was added to the culture so that the final inhibitor titer was 1 BU/ml, as indicated. The anti-FVIII ASC spots were detected as described in Figure 3C. **(A)** Representative pictures of the anti-FVIII ASC spots from the different groups. **(B)** The data in the histograms are expressed as mean ± SEM. **p < 0.01 compared to the rFVIII only group by the Student’s *t*-test.

For potential clinical translation of the BAR Treg approach, it is important to know whether the BAR Tregs would function properly in a primed host with pre-existing anti-FVIII inhibitors. Toward this goal, the inhibitor titer of pooled sera from FVIII-immunized E16 mice was determined using the modified Bethesda assay (data not shown), and A2-BAR human natural Tregs were generated as previously described (8). The FVIII-specific memory B cell suppression assay was carried out in the presence of the pooled sera diluted to a final inhibitor concentration of 1 BU/ml. The addition of human A2-BAR Tregs completely suppressed FVIII-specific ASC formation independent of the presence/absence of FVIII-inhibitory antibodies (Figure 5).

## Discussion

Anti-drug antibody formation to therapeutic FVIII is considered the most severe side effect in the treatment of HA patients, and FVIII-specific memory B cells are key targets toward the goal of eradicating inhibitors and establishing tolerance to FVIII (2). We report here that both mouse and human natural Tregs engineered to express the FVIII A2 domain (A2-BAR Tregs) effectively suppressed the activity of FVIII-specific memory B cells in an *in vitro* assay. To our knowledge, this is the first proof-of-principle demonstration that natural Tregs can be engineered to target antigen-specific memory B cells.

Although most inhibitors are against the functionally important A2 and/or C2 domain of FVIII, the anti-FVIII immune response is heterogeneous (20). Therefore, an important question to be answered regarding the BAR Treg approach is whether Tregs engineered to express a single domain of the antigen are sufficient to suppress inhibitor responses against epitopes located on other domains. Since the *in vitro* assay used in our study measures the activity of polyclonal FVIII-specific memory B cells, the complete suppression of anti-FVIII ASCs formation by A2-BAR human Tregs clearly suggests a beneficial bystander suppression effect by the BAR Tregs in the local milieu (Figure 5).

Two aspects of the BAR Treg-mediated suppression have been addressed in this study. First, expressing A2-BAR on Tregs was required for the suppressive activity. When A2-BAR Tcon cells were added, they were unable to suppress the activity of FVIII-specific memory B cells (Figure 3D). Second, A2-BAR Tregs acted on the FVIII-specific memory B cells in a cell contact-dependent manner, since the suppression was completely abolished when Tregs and responders were separated using a transwell setting (Figure 3E). However, the detailed mechanisms of action by FVIII A2-BAR Tregs remain to be determined in future studies.

The BAR used in the current study contains transmembrane and signaling domains CD28-CD3ζ of human origin. Such chimeric receptors were functional in our *in vitro* studies, as well as indicated by others (21, 22). However, human CD3 and CD28 components may eventually be immunogenic, which could prevent long-term *in vivo* following up of the adoptively transferred BAR Tregs in immunocompetent mice. Therefore, future *in vivo* studies of the effect of FVIII A2-BAR Tregs will utilize fully murine CD28-CD3ζ components.

One limitation of the BAR Treg approach is that fully differentiated plasma cells no longer express BCR, so they are not targeted by BAR Tregs, as would be expected. However, not all FVIII-specific plasma cells are long-lived. The short-lived ones may still be indirectly targeted since they may rely on specific memory B cells for replenishing.

Taken together, the present results show that using an established *in vitro* memory B-cell assay system, FVIII A2-BAR natural Tregs effectively suppressed the activity of FVIII-specific memory B cells. The suppression was contact-dependent and required the BAR receptor to be expressed on Tregs. Although detailed mechanisms are still to be elucidated, we believe these findings have important implications for potential clinical translation of this approach to reverse inhibitor responses in HA, as well as other anti-drug antibody responses.

## Data Availability Statement

All datasets generated for this study are included in the article/Supplementary Material.

## Ethics Statement

The studies involving human participants were reviewed and approved by the Uniformed Services University of the Health Sciences Institutional Review Board. Written informed consent for participation was not required for this study in accordance with the National Legislation and the Institutional requirements. The animal study was reviewed and approved by Institutional Animal Care and Use Committee at USUHS.

## Author Contributions

AP, SV, A-HZ, and DS designed the experiments. AP, SV, and A-HZ performed the experiments and analyzed the data. A-HZ and DS conceived the project and wrote the manuscript.

## Conflict of Interest

A-HZ and DS are inventors on a patent application for antigen-specific Tregs.

The remaining authors declare that the research was conducted in the absence of any commercial or financial relationships that could be construed as a potential conflict of interest.

## Supplementary Material

FIGURE S1 The depletion of CD138^+^ cells in splenocytes from FVIII immunized E16 mice by magnetic-activated cell sorting (MACS). The depletion was performed using anti-mouse CD138 MicroBeads (Miltenyi Biotech), per the manufacturer’s protocol. The cells before (pre-depletion), after depletion (post-depletion), as well as the depleted fraction were stained with anti-mouse antibodies for B220 and CD138. The dot plots shown were gated on singlets → size → viable cells.

## References

[1] Hoyer LW. Hemophilia A. *N Engl J Med.* (1994) 330:38–47. 8259143 10.1056/NEJM199401063300108

[2] Schep SJ Schutgens REG Fischer K Boes ML. Review of immune tolerance induction in hemophilia A. *Blood Rev.* (2018) 32:326–38. 10.1016/j.blre.2018.02.003 29482894

[3] Brackmann HH Gormsen J. Massive factor-VIII infusion in haemophiliac with factor-VIII inhibitor, high responder. *Lancet.* (1977) 2:933 10.1016/s0140-6736(77)90871-6 72276

[4] van Helden PM Kaijen PH Fijnvandraat K van den Berg HM Voorberg J. Factor VIII-specific memory B cells in patients with hemophilia A. *J Thromb Haemost.* (2007) 5:2306–8. 17958750 10.1111/j.1538-7836.2007.02736.x

[5] Hausl C Ahmad RU Sasgary M Doering CB Lollar P Richter G High-dose factor VIII inhibits factor VIII-specific memory B cells in hemophilia A with factor VIII inhibitors. *Blood.* (2005) 106:3415–22. 16091456 10.1182/blood-2005-03-1182 PMC1895061

[6] Hausl C Ahmad RU Schwarz HP Muchitsch EM Turecek PL Dorner F Preventing restimulation of memory B cells in hemophilia A: a potential new strategy for the treatment of antibody-dependent immune disorders. *Blood.* (2004) 104:115–22. 15001466 10.1182/blood-2003-07-2456

[7] Reipert BM Allacher P Hausl C Pordes AG Ahmad RU Lang I Modulation of factor VIII-specific memory B cells. *Haemophilia.* (2010) 16:25–34. 10.1111/j.1365-2516.2008.01962.x 20536983

[8] Zhang AH Yoon J Kim YC Scott DW. Targeting antigen-specific B cells using antigen-expressing transduced regulatory T cells. *J Immunol.* (2018) 201:1434–41. 10.4049/jimmunol.1701800 30021767 PMC6103823

[9] Parvathaneni K Scott DW. Engineered FVIII-expressing cytotoxic T cells target and kill FVIII-specific B cells in vitro and in vivo. *Blood Adv.* (2018) 2:2332–40. 10.1182/bloodadvances.2018018556 30232086 PMC6156881

[10] June CH O’Connor RS Kawalekar OU Ghassemi S Milone MC. CAR T cell immunotherapy for human cancer. *Science.* (2018) 359:1361–5. 10.1126/science.aar6711 29567707

[11] Zhao DM Thornton AM DiPaolo RJ Shevach EM. Activated CD4+CD25+ T cells selectively kill B lymphocytes. *Blood.* (2006) 107:3925–32. 16418326 10.1182/blood-2005-11-4502 PMC1895290

[12] Ludwig-Portugall I Hamilton-Williams EE Gottschalk C Kurts C. Cutting edge: CD25+ regulatory T cells prevent expansion and induce apoptosis of B cells specific for tissue autoantigens. *J Immunol.* (2008) 181:4447–51. 18802046 10.4049/jimmunol.181.7.4447

[13] Lim HW Hillsamer P Banham AH Kim CH. Cutting edge: direct suppression of B cells by CD4+ CD25+ regulatory T cells. *J Immunol.* (2005) 175:4180–3. 16177055 10.4049/jimmunol.175.7.4180

[14] Iikuni N Lourenco EV Hahn BH La Cava A. Cutting edge: regulatory T cells directly suppress B cells in systemic lupus erythematosus. *J Immunol.* (2009) 183:1518–22. 10.4049/jimmunol.0901163 19570829 PMC2730469

[15] Gotot J Gottschalk C Leopold S Knolle PA Yagita H Kurts C Regulatory T cells use programmed death 1 ligands to directly suppress autoreactive B cells in vivo. *Proc Natl Acad Sci USA.* (2012) 109:10468–73. 10.1073/pnas.1201131109 22689978 PMC3387068

[16] Qian J Collins M Sharpe AH Hoyer LW. Prevention and treatment of factor VIII inhibitors in murine hemophilia A. *Blood.* (2000) 95:1324–9. 10666206

[17] Bi L Lawler AM Antonarakis SE High KA Gearhart JD Kazazian HH Jr. Targeted disruption of the mouse factor VIII gene produces a model of haemophilia A. *Nat Genet.* (1995) 10:119–21. 7647782 10.1038/ng0595-119

[18] Zhang AH Skupsky J Scott DW. Effect of B-cell depletion using anti-CD20 therapy on inhibitory antibody formation to human FVIII in hemophilia A mice. *Blood.* (2011) 117:2223–6. 10.1182/blood-2010-06-293324 21160036 PMC3062330

[19] Thornton AM Korty PE Tran DQ Wohlfert EA Murray PE Belkaid Y Expression of Helios, an Ikaros transcription factor family member, differentiates thymic-derived from peripherally induced Foxp3+ T regulatory cells. *J Immunol.* (2010) 184:3433–41. 10.4049/jimmunol.0904028 20181882 PMC3725574

[20] Lollar P. Pathogenic antibodies to coagulation factors. Part one: factor VIII and factor IX. *J Thromb Haemost.* (2004) 2:1082–95. 15219191 10.1111/j.1538-7836.2004.00802.x

[21] Wang LC Lo A Scholler J Sun J Majumdar RS Kapoor V Targeting fibroblast activation protein in tumor stroma with chimeric antigen receptor T cells can inhibit tumor growth and augment host immunity without severe toxicity. *Cancer Immunol Res.* (2014) 2:154–66. 10.1158/2326-6066.CIR-13-0027 24778279 PMC4007316

[22] Festag MM Festag J Frassle SP Asen T Sacherl J Schreiber S Evaluation of a fully human, hepatitis B virus-specific chimeric antigen receptor in an immunocompetent mouse model. *Mol Ther.* (2019) 27:947–59. 10.1016/j.ymthe.2019.02.001 30852138 PMC6520494

