# Supplementary figures and images for: Suppression of FVIII-Specific Memory B Cells by Chimeric BAR Receptor-Engineered Natural Regulatory T Cells
Source: Front Immunol. 2020 Apr 21;11:693. doi: 10.3389/fimmu.2020.00693 (PMC7186411; doi:10.3389/fimmu.2020.00693)

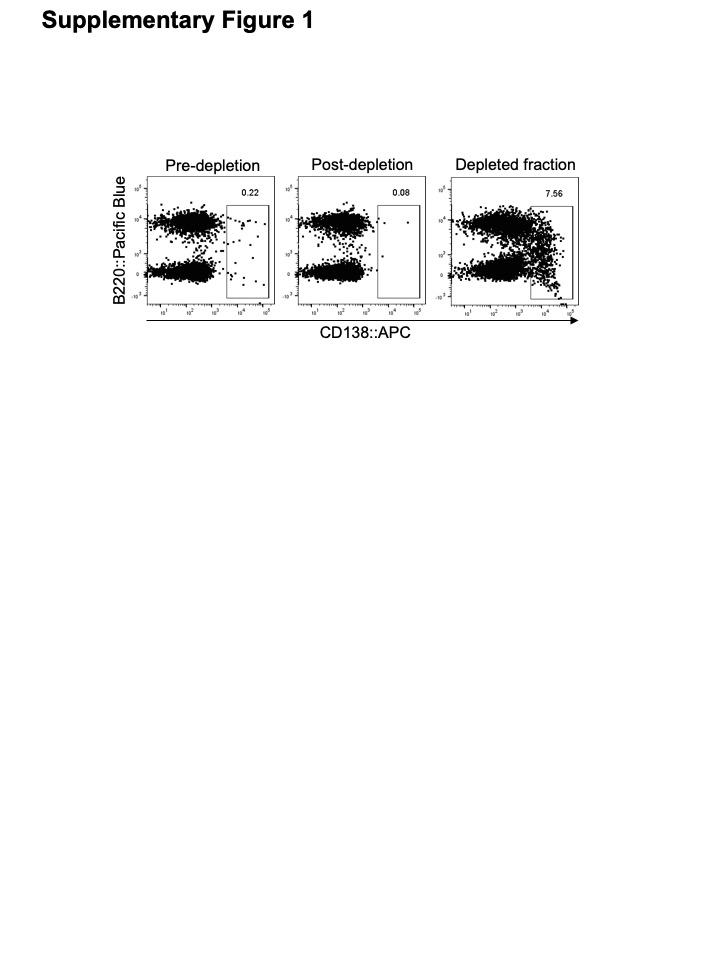

Supplement: FIGURE S1 — The depletion of CD138+ cells in splenocytes from FVIII immunized E16 mice by magnetic-activated cell sorting (MACS). The depletion was performed using anti-mouse CD138 MicroBeads (Miltenyi Biotech), per the manufacturer’s protocol. The cells before (pre-depletion), after depletion (post-depletion), as well as the depleted fraction were stained with anti-mouse antibodies for B220 and CD138. The dot plots shown were gated on singlets → size → viable cells. [file Image_1.jpeg]
